# Head-tracking of freely-behaving pigeons in a motion-capture system reveals the selective use of visual field regions

**DOI:** 10.1038/s41598-022-21931-9

**Published:** 2022-11-09

**Authors:** Fumihiro Kano, Hemal Naik, Göksel Keskin, Iain D. Couzin, Máté Nagy

**Affiliations:** 1 Centre for the Advanced Study of Collective Behaviour, University of Konstanz, Universitätsstraße 10, 78464 Konstanz, Germany; 2 Department of Collective Behaviour, Max-Planck Institute of Animal Behavior, Konstanz, Germany; 3 Department of Ecology of Animal Societies, Max-Planck Institute of Animal Behavior, Konstanz, Germany; 4 Computer Aided Medical Procedures, Teschnische Universiät Munchen, Munich, Germany; 5 Department of Biology, University of Konstanz, Konstanz, Germany; 6 MTA-ELTE Lendület Collective Behaviour Research Group, Hungarian Academy of Sciences, Budapest, Hungary; 7 Department of Biological Physics, Eötvös Loránd University, Budapest, Hungary

**Keywords:** Animal behaviour, 3-D reconstruction

## Abstract

Using a motion-capture system and custom head-calibration methods, we reconstructed the head-centric view of freely behaving pigeons and examined how they orient their head when presented with various types of attention-getting objects at various relative locations. Pigeons predominantly employed their retinal specializations to view a visual target, namely their foveas projecting laterally (at an azimuth of ± 75°) into the horizon, and their visually-sensitive “red areas” projecting broadly into the lower-frontal visual field. Pigeons used their foveas to view any distant object while they used their red areas to view a nearby object on the ground (< 50 cm). Pigeons “fixated” a visual target with their foveas; the intervals between head-saccades were longer when the visual target was viewed by birds’ foveas compared to when it was viewed by any other region. Furthermore, pigeons showed a weak preference to use their right eye to examine small objects distinctive in detailed features and their left eye to view threat-related or social stimuli. Despite the known difficulty in identifying where a bird is attending, we show that it is possible to estimate the visual attention of freely-behaving birds by tracking the projections of their retinal specializations in their visual field with cutting-edge methods.

## Introduction

Both humans and nonhuman animals actively move their heads and eyes to retrieve information to guide natural tasks, such as moving around obstacles, searching for resources, being vigilant to potential risks, and predicting and remembering objects, patterns, and events^1–4^. Consequently, observing spontaneous head and eye movements of animals has been a common approach in various research fields to examine how individuals exert perception and cognition to navigate physical and social environments (e.g.^5–8^). However, as the quantitative tracking of freely-behaving animals’ head and eye movements is technically challenging, the development of this research field is closely related to the availability of relevant technologies. Recent advances in technologies, including the miniaturization of head-mounted devices and the emergence of contact-free imaging techniques, have made head- and eye-tracking of freely-behaving animals increasingly more accessible to researchers (e.g.^9–12^). Notably, recent detailed and simultaneous tracking of the body postures of all animals in a group (a fish school) enabled researchers to reconstruct a two-dimensional representation of all members’ visual fields and the group’s visual sensory network^13,14^. In so doing, they revealed how individuals dynamically influence the group’s movement decisions through such an information network.

One key feature of head and eye-tracking is its ability to infer where a subject is overtly attending at a given moment. In primates, this type of inference is largely possible because, when an individual primate attends to an object, this individual typically coordinates the movements of the two eyes and orients the fovea, the most visually sensitive spot in the retina, of each eye to a visual target for a somewhat lengthy time (called “fixation”). In birds, however, previous studies reported that there are fundamental difficulties in inferring where a subject is looking in a manner similar to primate eye-tracking^15,16^. There are three main reasons for this. First, although birds typically have specialized regions for high visual sensitivities (called foveas, visual streaks, and areas^17^) unlike primates, many bird species have more than one retinal specialization in each eye; for example, one central fovea and one temporal area. Therefore, in such species, we should assume three “lines of gaze”: one projecting frontally from the two eyes, and two projecting laterally. Second, in birds, the configurations of the retina (e.g., the location of foveas/areas in the retina) and visual field (e.g., the size of binocular and blind regions in the visual field) are known to differ substantially among species depending on the species’ niche^17–19^. An individual bird is thus expected to orient its head and eyes to a visual target in a unique manner depending on its species-unique configuration of the retina and visual field^4^. Third, although the visual orienting response of birds is directly observable—typically as a succession of head saccades (a quick ballistic movement of the head) similar to primate eye saccades—they do not seem to fixate or pursue a visual target for a lengthy time with their heads/eyes as primates do^9,15,16,18^; they have thus been described as being “extremely ‘shifty-headed’ ”^15^.

However, it should be possible to infer, at least with some accuracy, where a bird is looking if we know the properties of the visual system of its species. Birds indeed move their head and eyes in a predictable manner during various natural behaviors. For example, when foraging on the ground, birds are less vigilant when they are in head-down postures compared to when they adopt head-up postures. This is because their blind regions in the visual field generally project towards the sky in head-down postures^20,21^ (but see^4^). Some species of birds use particular head angles to view a visual target with their retinal specializations^15,16,22–27^; for example, a finch trying to peck a grain shifts its head sideways (azimuth of 60° away from the beak) to view the grain by its fovea^23^; a tern trying to catch a crab on the ground uses its frontal visual field to pursue the prey while occasionally shifting its head sideways (azimuth of 45° away from the beak) to scrutinize the prey with its fovea^16^; a falcon flying toward the prey follows a curved flight path and keeps sideways views of the prey to try to maintain it within its fovea (azimuth of 40° away from the beak)^24^.

Recent technological advances allow us to perform head/eye-tracking of freely behaving birds with high spatiotemporal accuracy, although the applicability of such techniques depends on the species and behaviors tested in the studies. For example, a head-mounted eye-tracker was employed to record both head and eye movements of relatively large birds, such as peafowls and chickens, when they were freely behaving on the ground^10,27–29^. Given that eye movement is confined, at least in some species, to only a few degrees^30–34^ (but see^28,35^), simple head-tracking can also be informative; for example, a head-mounted miniature camera was used to record visual pursuit of prey in hawks and falcons^24–26^; a head-mounted Inertial Measurement Unit (IMU) sensor was used to record head movements of homing pigeons during navigation and flocking^11,36,37^. However, while essential for field studies, these head-mounted devices are currently relatively large, especially for small-sized birds.

Under circumstances where birds move in a confined or predictable location, such as in a lab or near a feeder and nest in the wild, it is possible to film them from one, or multiple, perspectives using standard cameras. A conventional method is to analyze the two-dimensional (2D) head orientations of birds in each camera frame-by-frame (e.g.^15,16,38^). However, this method is not able to analyze the 6 degrees-of-freedom (DOF) movements of birds’ heads. A potential, emerging alternative technique is using multi-array camera images and deep learning algorithms to estimate 3-dimensional (3D) body postures including 6 DOF head movements^39,40^ (also see^41–43^), but the accuracy and applicability of this approach have not yet been thoroughly explored in birds. Another technique, and the one that we will adopt here, is to use a motion-capture system that can record the 3D coordinates of small reflective markers attached to a bird’s head with high spatiotemporal resolutions. Previous studies have employed this method to study pigeons’ visual perception and 3D head orientations during perching (head stabilization)^44^, pecking (beak control)^45^, and walking (head bobbing)^46^. However, its use has been limited to relatively small laboratory spaces and the analysis of these relatively small-scaled individual behaviors.

Here, we employed a motion capture system to track precise body postures of pigeons (*Columba livia*) and then applied this information to reconstruct their head-centric views during their free behaviors. Our motion-capture system is designed to track the detailed movements of multiple freely-behaving/interacting animals within a relatively large 3D space (15w × 7d × 4h m, in width, depth, and height)^47^. Our first aim was to reconstruct the head-centric view, or the visual field, of pigeons using our custom head-calibration methods. Pigeons were chosen because their visual system is well-characterized (see below). Our second aim was to test whether, when pigeons are presented with an attention-getting visual target from various 3D locations, they use their visual fields in a predictable manner. Our final aim was to employ the obtained results to construct the visual-field model that can be used to infer (a few candidate) locations where a pigeon is attending during its free behaviors.

Our general prediction was that pigeons will use the projections of their retinal specializations to assess the presented stimuli. Specifically, pigeons have two retinal specializations in each eye: (1) the central fovea (or also called area *centralis*, located centrally in the retina), which is projected laterally and horizontally into the monocular visual field, and (2) another visually sensitive spot, known as the “red area” (named based on its reddish appearance in the retina, and located dorsally in the retina), which is projected more forward and downward, and broadly into the lower-frontal visual field^48,49^. The central fovea has a higher visual acuity and motion sensitivity compared to the other parts of the retina and is optimized to view a distant and/or fast-moving visual target^22,50,51^. There is evidence showing that pigeons are myopic (near-sighted) in the lower-frontal visual field^52–54^ and the use of the lower-frontal visual field seems to be largely restricted to food search and food pecking^45,55^. We thus predicted that pigeons primarily use the lower-frontal visual field to view a nearby grain on the ground and the central foveas to view relatively distant and/or fast-moving visual targets.

Moreover, we predicted that pigeons have different eye preferences for distinct object types (although they generally should use either eye to view any object). Pigeons and chickens are generally better at tasks requiring them to attend to the detailed features of stimuli, such as distinguishing grain from inedible items, when they use their right eye (thus the left-brain hemisphere) as compared to when they use their left eye ^56–58^. Pigeons tend to follow conspecifics on their left side during coordinated flights^59^. Chickens interact more often with a conspecific when they use their left eye (thus the right-brain hemisphere) as compared to when they use their right eye^60^. Moreover, chickens and several other species of birds prefer to use their left eye when they are attending to a threat^61–63^ (but see^64^). We thus predicted that pigeons generally have a left eye preference for social and threatening stimuli and a right eye preference for non-threatening, non-social, and inedible stimuli when those stimuli and food grains were alternately presented to the birds (namely, when the birds need to attend to the detailed features of stimuli).

Lastly, pigeons move their eyes relative to their heads, which is expected to add errors in our data because our system does not track their eye movement. Previous studies of pigeons have shown that the majority of eye movements occur in conjunction with head movements^30,31,33,34^. Eye movement can reach up to as much as 26^30,65^, but the majority of eye movements are typically smaller than 5°^30,31^. Furthermore, the eye-in-orbit locations of a bird typically follow a Gaussian distribution^29^. Thus, in our experiments, eye movement is expected to cause Gaussian-like errors, and a majority of such errors fall within a few degrees.

## Results

**Figure 1:**
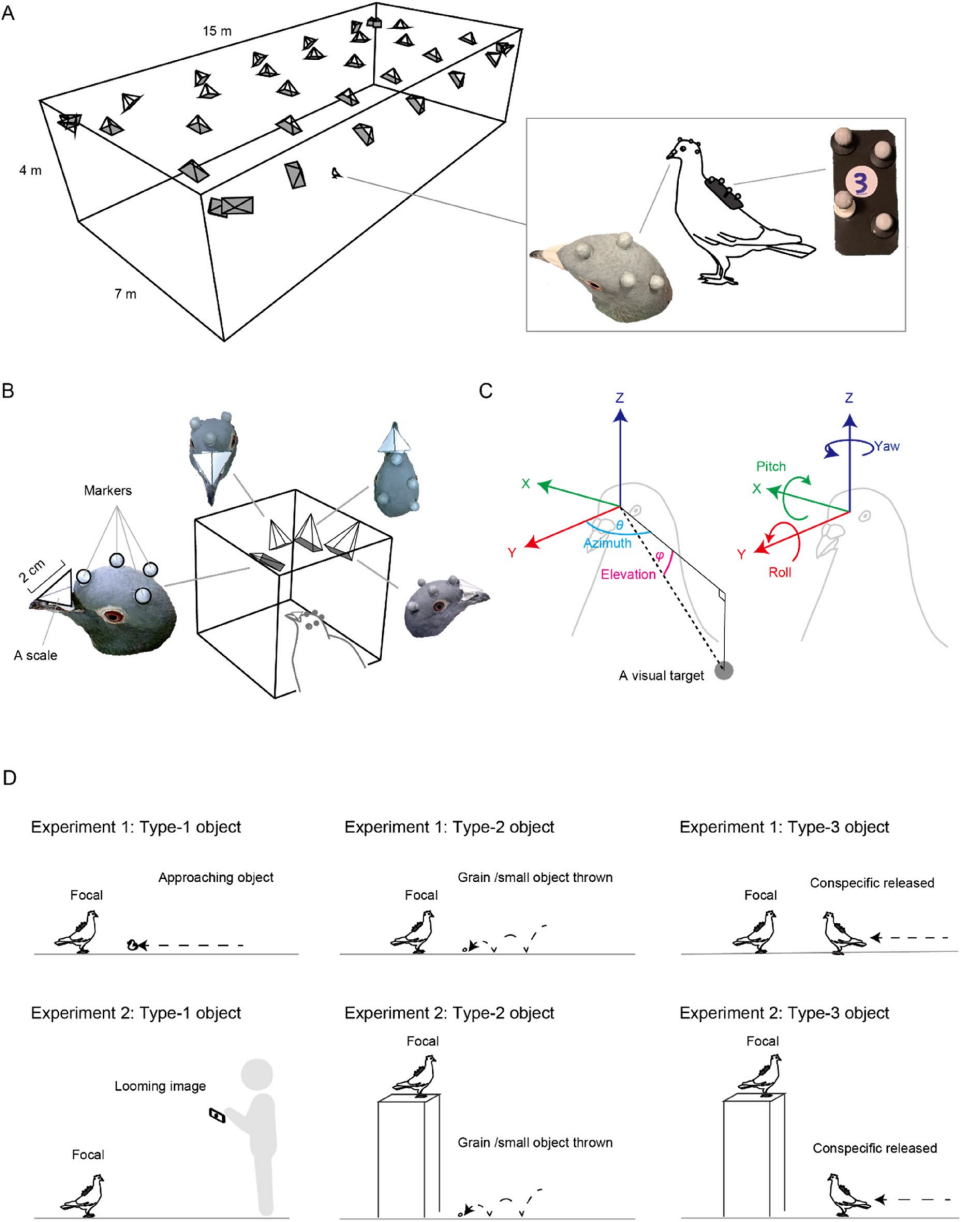
(**A**) A motion capture system (15 m × 7 m × 4 m) with 30 infrared and 4 optical cameras. The inset shows a pigeon wearing mo-cap markers attached to its head and back. (**B**) The head-calibration system with 4 optical cameras to identify the 3D coordinates of head markers, eyes, and beak tip. (**C**) Reconstructed head-centric coordinate system. The 3D position of a visual target was described in a Polar coordinate system (azimuth and elevation). (**D**) Experimental procedures. Visual targets were either medium-sized approaching objects or looming images (Type-1; moderately threatening stimuli), small-sized objects thrown to pigeons alternately with food grains (Type-2; detailed stimuli), and conspecific individuals (Type-3; social stimuli). Experiment 1 presented the visual targets on the ground when the focal birds were also on the ground, and Experiment 2 presented them either above or below the focal birds’ heads.

A motion-capture (mo-cap) system was employed to track the 3D coordinates of reflective markers attached to the head and back of pigeons (Fig. 1A)^47^. Using our custom-developed head calibration system (Fig. 1B and Fig. S1), we constructed the head-centric coordinate system from the 3D coordinates of the eyes and beak tip, where the rotation of the coordinate system can be described as yaw, roll, and pitch, and the local coordinate of a visual target can be described as azimuth and elevation (Fig. 1C).

To examine pigeons’ use of different regions of visual fields, we presented three types of attention-getting objects from various 3D locations across two experiments (Fig. 1D and Fig. S2). We prepared three types of visual stimuli to induce distinct behavioral contexts by presenting distinctive types of stimuli. Visual stimuli consisted of: Type-1, moderately threatening stimuli (either medium-sized approaching objects or looming images); Type-2, detailed stimuli (small-sized objects thrown to pigeons alternately with food grains, thus that pigeons need to attend to the detailed features of stimuli); and Type-3, social stimuli (conspecific individuals). Experiment 1 tested ten birds and presented the three types of visual targets on the ground when the focal birds were also on the ground. Experiment 2 tested an additional ten birds and varied the heights of the presented stimuli; we presented Type-1 stimuli above the focal birds’ heads (the focal birds were on the ground, and the stimulus was presented at an elevated location), and Type-2 and 3 stimuli below the focal birds’ heads (the focals were on an elevated location, and the visual target was presented on the ground). We thus had six presentation conditions across the two experiments (20 birds in total; see Table S1 for the summary of these conditions). We used this variation of presentation conditions in our analysis to elucidate a general pattern of pigeons’ visual-field use across the conditions in our first analysis (by including this variation as a random factor in the statistical model) and also to use specific conditions to examine several focused questions in our subsequent analyses (specifically with regard to the differential use of distinct retinal specialization in food search and the laterality of eye use according to the object types).

In general, pigeons typically moved away from Type-1 (moderately threatening) stimuli in Experiments 1 and 2, while they approached Type-2 (detailed) and Type-3 (conspecific) stimuli in Experiment 1 (i.e., where the pigeons and objects were not separated by the elevation of the table as in Experiment 2). When they were presented with a Type-2 stimulus (a small marker attached to a peanut) at a distance (in Experiment 1), they often first oriented their lateral visual field to the object, occasionally then approached and attempted to peck it using their frontal visual field. While they oriented their lateral visual fields typically only once when presented with Type-1 or Type-2 stimuli, they repeatedly oriented both left and right lateral visual fields in succession when presented with Type-3 (conspecific) stimuli (in Experiments 1 and 2). See Video S1 for examples of these behaviors.

We presented the visual stimuli to both single and multiple birds across the repeated presentations of the stimuli within a day (Table S2) and included both types of recordings in our analyses because both yielded largely similar results. To capture stimulus-driven attention in birds, from the movement data of the presented object, we extracted the moments in which the object just appeared in the pigeons’ visual field, as the sudden appearance of a visual stimulus generally evokes birds’ orienting responses ^22,27^; specifically, we focused on the moments in which a thrown/slid object (Type-1 or Type-2) recently stopped at a visible location (0–2 s. since the object stopped moving), a looming image (Type-1) was presented to the focal birds, and a conspecific (Type-3) appeared in the motion-capture room while the focal bird was already present (see “Method” for the details).

**Figure 2:**
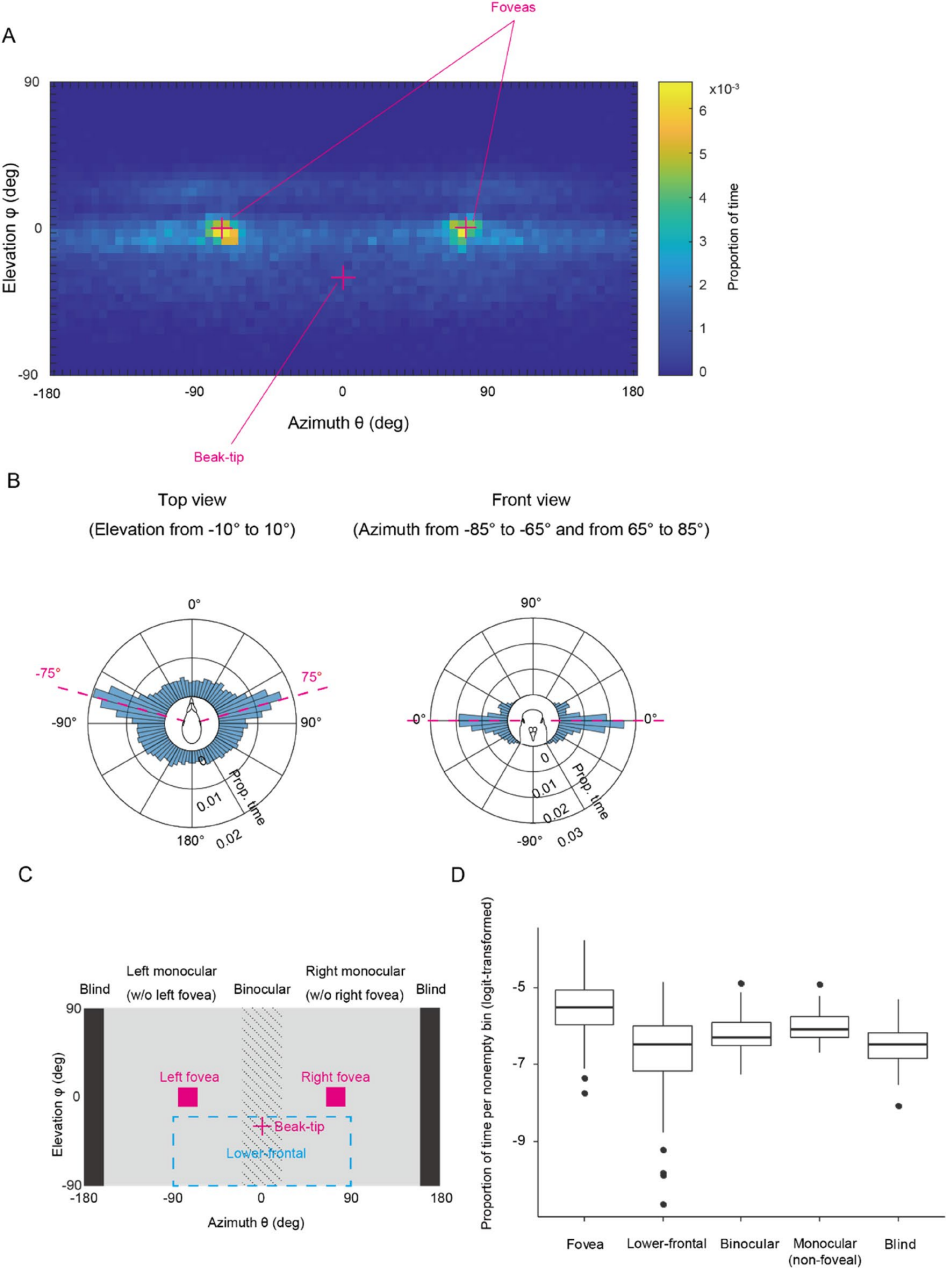
(**A**) The distribution of visual targets in pigeons’ visual field. Each bin was sized 5° × 5° and indicate the mean proportion of time during which visual targets were observed in this bin across 2 experiments presenting 3 object types (i.e., the mean of 6 heatmaps). The cross marks indicate the projections of foveas (an azimuth of ± 75° and an elevation of 0°) and beak-tip (an azimuth of 0° and an elevation of −30°). B. The same data in polar histograms while restricting (for the visualization purpose) the distribution to a range including the elevation from −10° to 10° (left panel, the top view), and the azimuth from −85° to −65° (left of the bird) and 65° to 85° (right of the bird) (right panel, the front view). (**C**) Our operational definition of foveal, lower-frontal, binocular, monocular (non-foveal), and blind regions. (**D**) Proportion of time during which visual targets were observed in each non-empty bin of each visual field region (logit-transformed). Box plots show the median, interquartile range (IQR), and 1.5 × IQR with outliers plotted individually.

To estimate regions of the visual field that the birds most frequently used across all presentation conditions, we pooled the data from all birds for each condition (6 conditions in total) and plotted the distribution of visual targets in the visual field as a heatmap (Figure S3A). We then derived an average heatmap by averaging all 6 heatmaps per bin (Fig. 2A). The color (value) of each bin (5 × 5°) in the heatmap indicates the proportion of time during which (or frames in which) the visual targets were observed to be in the region of the visual field associated with this specific bin. Figure 2B shows the same data as polar histograms. We found two clear peaks at an azimuth of ± 75° and an elevation of 0° (the horizon), which approximately matches the previously reported locations of the foveal projections (or the optic axes close to them)^48,49,66,67^.

To quantify the birds’ use of distinct regions of visual fields, we defined an operational range for each visual field region (Fig. 2C). We defined the projections of foveas as being centered at an azimuth of ± 75° and an elevation of 0° (i.e., the horizon) based on both previous studies (which slightly differ between the studies^48,49,66,67^) and our above observations (Fig. 2A,B). We added a margin of 10° on both positive and negative sides of these centers, again based on both previous studies and our above observations (Fig. 2A,B), to loosely accommodate both certain degrees of eye movement around foveas (typically reported as up to 5°^30,31^) and potential errors in estimating the head orientations in our system. Additionally, we defined the lower-frontal region to include the azimuth from −90° to 90° and the elevation from −20° to −90°; i.e., around and below the beak (the eye-beak line was about −30°; see Fig. S4), as previous studies have indicated that the red area (the secondary sensitive spot in their retina) projects broadly into the lower-monocular visual field^48,49^. Moreover, we defined the binocular region to include the azimuth from −20° to 20°, which accommodates all previously reported values of binocular overlap (which differ between studies^49,67,68^). Finally, we defined the blind region to include the azimuth from 160° to 180° and −160° to −180° following a previous study^69^ and the monocular regions as the remaining regions (not including foveal, binocular, and blind regions). Note that the lower-frontal region overlaps with both binocular and non-foveal monocular regions in these definitions.

We then calculated the mean bin value of each visual field region in each condition for each bird; for this calculation of the mean, we included only nonempty bins (in which at least one visual target fell) given that a large portion of the visual field was not used by birds in our experiments. After logit-transforming the mean bin values (i.e., the proportion of time per nonempty bin) to achieve normally distributed residuals^70^, we performed a linear mixed model (LMM) with the mean bin value as a response, the visual field region as a fixed factor, and the focal subject as a random factor. Additionally, we included our six presentation conditions (which varied in object types and presentation heights) as a random factor (because, as mentioned above, we aimed to reveal the birds’ use of visual fields given these variations). We found a significant effect of the visual field region (likelihood ratio test, *χ*^2^(4) = 20.85, *P* = 0.00034); the visual targets fell most frequently in the foveal bins and least frequently in the blind and lower-frontal bins (Fig. 2D). We observed a generally similar pattern across different presentation conditions (Fig. S3). Although there was a condition identified as an influential case in our analysis (Experiment 2 presenting Type-1 objects), removing this condition yielded the same results.

In our additional analysis (Fig. S5), we show that pigeons oriented their foveas to the thrown/slid objects (Type-1 and 2 in Experiment 1, and Type-2 in Experiment 2) mainly just after those objects landed on (0–2 s. after those objects stopped their movements), but this response diminished as time progressed further, indicating that the pigeons actively adjusted their head orientations to foveate those visual targets at specific moments.

**Figure 3:**
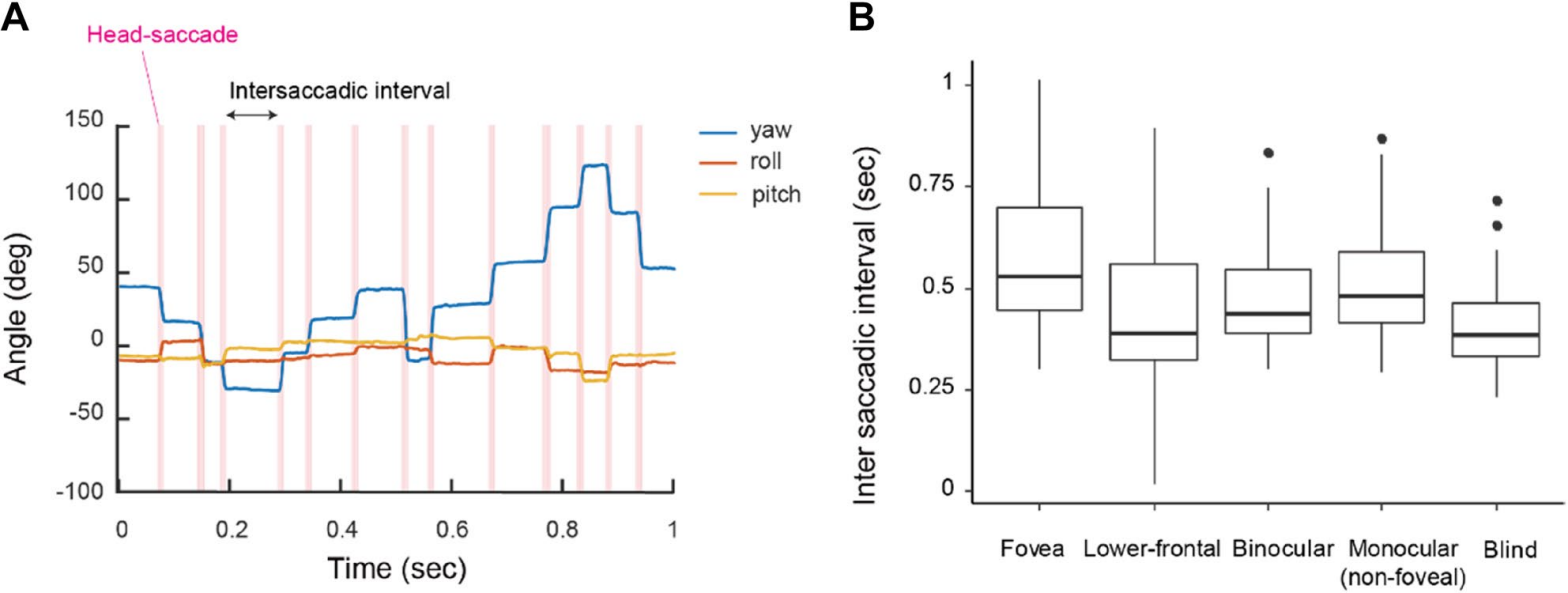
(**A**) Example of head movement in yaw, roll, and pitch. Saccades are highlighted in magenta. (**B**) Intersaccadic intervals (s) when a visual target was in each visual field region. Box plots show the median, interquartile range (IQR), and 1.5 × IQR, with outliers plotted individually.

To test whether birds “fixated” their head on the visual targets, we examined the intervals between their head saccades (Fig. 3A; also see Fig. S6) and tested whether the intersaccadic interval was longer when the visual target was in a certain visual field region, particularly foveas. An LMM with the intersaccadic interval as a response, the visual field region as a fixed factor, and the focal subject and presentation condition as random factors revealed a significant effect of the visual field region (likelihood ratio test, *χ*^2^(4) = 11.26, *P* = 0.024); the intersaccadic interval was longer when the birds used the foveal regions compared to the other visual field regions (Fig. 3B).

**Figure 4:**
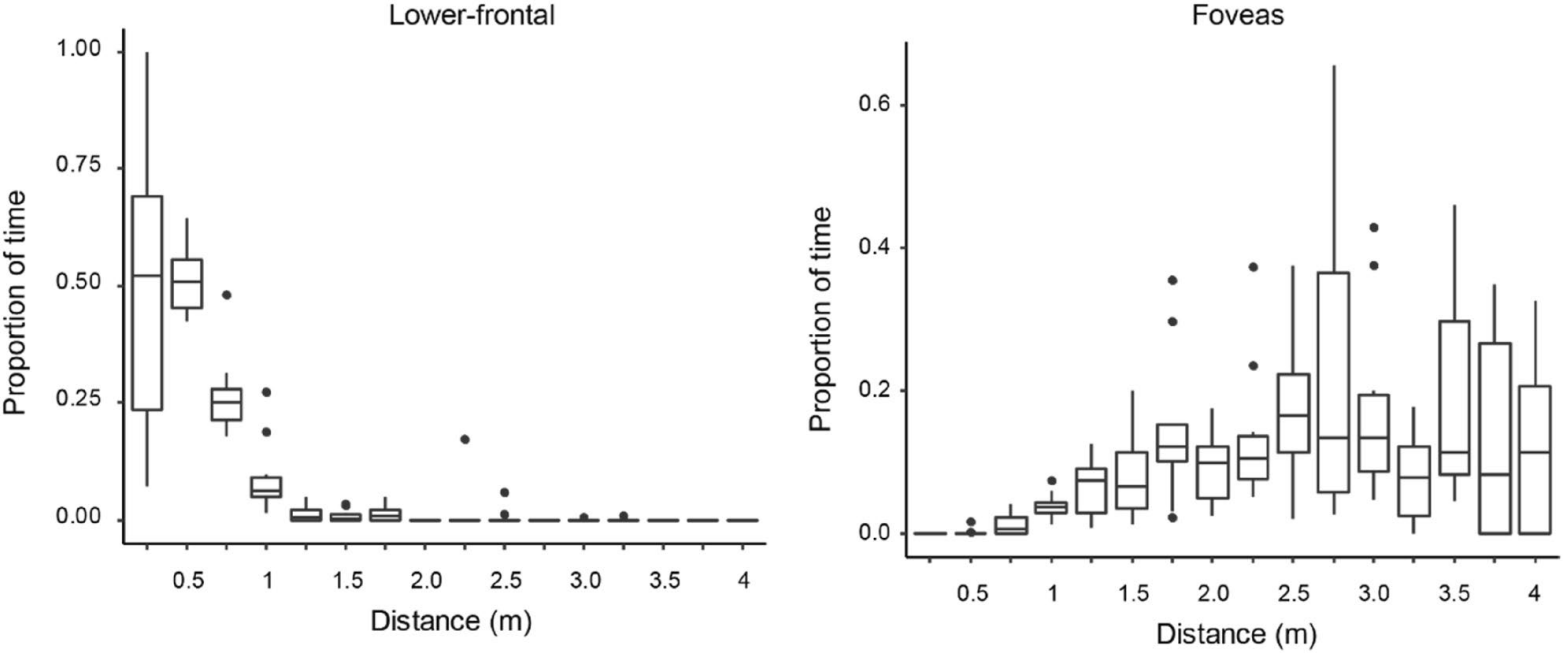
Proportion of time per bird during which visual targets were observed respectively in the foveal and lower-frontal regions in Experiment 1 presenting Type-2 (detailed) targets. Box plots show the median, interquartile range (IQR), and 1.5 × IQR, with outliers plotted individually.

Although the overall pattern showed that the birds primarily used their foveas, but not their lower-frontal visual fields, we also examined whether they used their lower-frontal visual field under a specific condition, particularly when they assessed visual targets at close distances; this is because previous studies have shown that pigeons use their lower-frontal visual fields when searching for food grains and attempting to peck a grain or small object at a close distance^49,55^. We thus focused on the condition where we presented Type-2 (detailed) objects in Experiment 1 (Fig. 1D) because this was the only condition where visual targets fell at both close to far distances from the pigeons (Fig. S2). We quantified pigeons’ differential use of foveal and lower-frontal regions as a function of distance to the visual targets. More specifically, we binned the distance with 0.25-m intervals, up to 4 m, and calculated the proportion of time during which visual targets were observed in the foveal and lower-frontal regions at each distance bin for each bird (Fig. 4). To test the effect of distance on the birds’ use of foveal and lower-frontal regions, we performed a binomial generalized linear mixed model (GLMM) with the number of frames in which the visual target was hit or missed in each region as a response (i.e., the proportion of time), the distance, the visual field region, and their interaction as fixed factors, and the subject as a random factor.

We found a significant interaction effect between the two fixed factors (Likelihood ratio test, *χ*^2^(15) = 253.36, *p* < 10^–15^), revealing that the birds decreased the use of the lower-frontal region while they increased the use of the foveal region as the distance from the visual targets increased. It should be noted that we could observe this distance-related pattern only under this particular presentation condition because our birds rarely allowed the other types of stimuli (Type-1 moderately-threatening objects and Type-3 conspecifics) to be as close as the Type-2 (detailed) stimuli in Experiment 1 (Fig. S2), and they predominantly used their foveas, but not their lower-frontal visual fields, when they were presented with Type-2 stimuli at a distance and below their heads in Experiment 2 (Fig. S3). In additional analyses, we confirmed that pigeons employed retinal specializations predominantly shortly after the thrown objects stopped moving (0–2 s. after stopping; Fig. S5), but this response diminished as time progressed further. Moreover, we confirmed that the birds used the broad area of lower-frontal regions when the visual targets were moderately close (< 25 cm), likely when the birds were searching for grain in a head-down posture^48,49^ but used the visual fields only around the principal axis of the beak when the visual targets were even closer (< 15 cm), likely when the birds attempted to peck it^55^ (Fig. S7).

**Figure 5:**
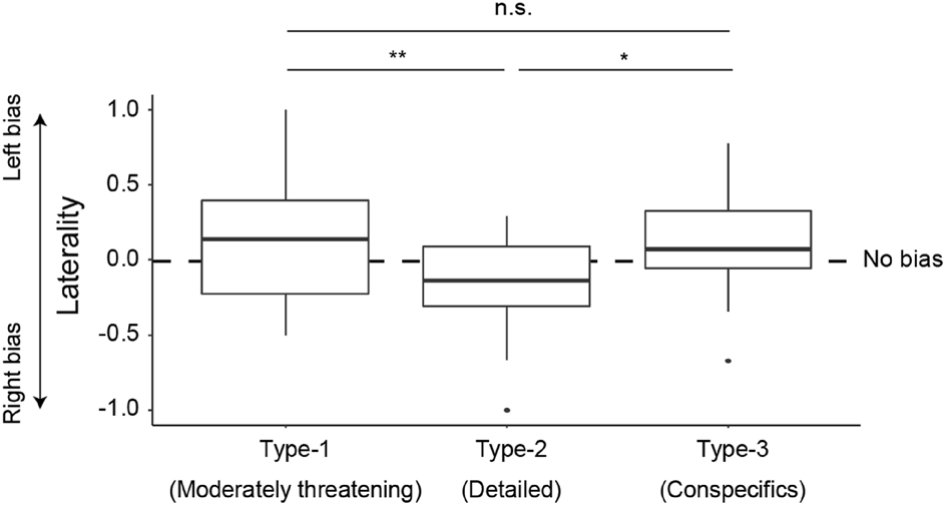
Laterality of foveal use in response to the 3 types of visual targets. Laterality was defined as (t_left_ – t_right_)/(t_left_ + t_right_), where t denotes looking time (in the foveas). Box plots show the median, interquartile range (IQR), and 1.5 × IQR, with outliers plotted individually. Asterisks indicate significance (*p < 0.05, **p < 0.01) in the posthoc analyses (*n.s.* no significance). No significant difference was found from the chance level (no left/right bias).

Finally, we examined the laterality of eye use in response to the 3 types of objects (Fig. 5). We calculated the laterality score as a bias in birds’ use of their left versus right foveas in all conditions; that is, the laterality score was defined as (t_left_ – t_right_)/(t_left_ + t_right_), where t denotes looking time. GLMM with the object type as a fixed factor, and the subject as a random factor, and the laterality score as response revealed a significant main effect of object type (*χ*^2^(2) = 9.36, *p* = 0.0093). To examine this main effect further, we performed posthoc pairwise paired t-tests and found that pigeons showed a stronger left bias when attending to Type-1 (moderately threatening) and Type-3 (social) targets compared to when attending to Type-2 (detailed) targets [Type-1 vs. Type-2: *t*(19) = 3.42, *p* = 0.003, *d* = 0.76; Type-2 vs. Type-3: *t*(19) = 2.30, *p* = 0.033, *d* = 0.51; Type-1 vs. Type-3: *t*(19) = 0.38, *p* = 0.71, *d* = 0.09]. Posthoc one-sample t-tests testing a bias in birds’ responses to each object type did not reveal any significance [Type-1: *t*(19) = 1.60, *p* = 0.13, *d* = 0.36; Type-2: *t*(19) = 2.00, *p* = 0.060, *d* = 0.45; Type-3: *t*(19) = 1.31, *p* = 0.20, *d* = 0.29].

## Discussion

Using a motion-capture system with custom head-calibration methods, we reconstructed freely behaving pigeons’ head-centric view in a large 3D space. We presented pigeons with 3 types of attention-getting objects (Type-1, moderately threatening, Type-2, detailed, Type-3, social) from various 3D angles and examined how they employed different regions of their visual field to assess those objects. The distribution of visual targets in the head-centric coordinate system of pigeons indicated that they predominantly used their foveas to view visual targets. Our additional time-centric analysis (Fig. S5) indicated that they actively adjusted their head orientations to foveate visual targets at specific moments. We also found that pigeons predominantly used foveas relatively independently of the types and relative locations of visual targets. However, in one specific condition where we presented a looming stimulus above the birds’ head, they positioned the stimuli in both foveal and non-foveal regions (and less frequently in the blind region); it requires further examinations to clarify whether this result is simply related to procedural issues or the birds’ specific visual strategy for such (overhead, potential risky) stimuli.

We also found that the inter-saccadic interval was significantly longer when they used their fovea to view visual targets compared to when they used other parts of their visual field. This result suggests that, in contrast to what has been previously assumed^15^, pigeons exhibit “fixation” on visual targets (for a relatively lengthy time), likely facilitating a more effective examination of objects via their foveas.

Moreover, we found that pigeons rarely used their foveas to view a nearby object but used their lower-frontal visual fields to do so. This result is consistent with previous studies showing that pigeons are myopic in their lower-frontal visual field^53^ and that their visual acuity increases with distance in the lateral visual field (including foveas), and decreases with distance in the lower-frontal visual field^50^. One previous study showed that body-restrained pigeons viewed a fast-moving object with the lateral visual field (including foveas) and a slowly-moving or stationary object with the frontal visual fields (including upper/lower binocular regions) independently of the relative distances to the visual targets^22^. By contrast, in our study, we found that freely-moving pigeons predominantly used their foveas to view slowly-moving or stationary objects/conspecifics and that the relative distances of visual targets critically affected their use of foveal and lower-frontal regions. Although further studies are necessary to understand this discrepancy, procedural differences, such as the body-restraint condition, may have affected the results differently in this and previous studies; pigeons of the previous study might have perceived the presented stationary stimuli differently, for example, as a perch where they could land^71^.

We also found that the laterality of foveal use relates to the type of presented object, largely consistent with the previous studies^56–64^. Our pigeons were biased towards using their left eye when viewing Type-1 (moderately threatening) and Type-3 (conspecific) stimuli, compared to when viewing Type-2 (detailed) objects. However, the observed laterality did not significantly differ from chance level (equal use of left and right eye) in their responses to each object type. It should be noted that these observed relatively weak eye preferences are largely consistent with the previous studies which directly observed the birds’ eye or side preferences across various behavioral contexts^59,62–64^. A more common, and possibly more robust, method used to examine pigeons’ and chickens’ visual lateralization is the comparison of their task performances (e.g., in the pebble-grain task) between the conditions where a different eye of birds is occluded^56–58,60,61^. Our results minimally show that the laterality of their eye preference differs across behavioral contexts in pigeons. Our results also suggest that visual lateralization could be examined in this species, at least to some extent, by directly observing their eye preferences with our head-tracking methods.

Although a key advantage of using a motion-capture system is its ability to track 3D coordinates of reflective markers in high tracking accuracy, key shortcomings are the necessity of attaching small markers to the birds and also the time and cost needed to establish the motion-capture system. An alternative method is using multi-array cameras and deep-learning system to achieve markerless tracking of birds’ postures^39,40^ (also see^41–43^). Although the markerless tracking technique should be applicable in a wider variety of study situations as compared to the motion-capture technique, it is currently not compelling with the motion-capture technique in terms of tracking accuracy and robustness. Conversely, however, the motion-capture dataset can be used as ground truth to train the deep-learning system and help it to achieve higher tracking accuracy and robustness in future studies^41^.

**Figure 6:**
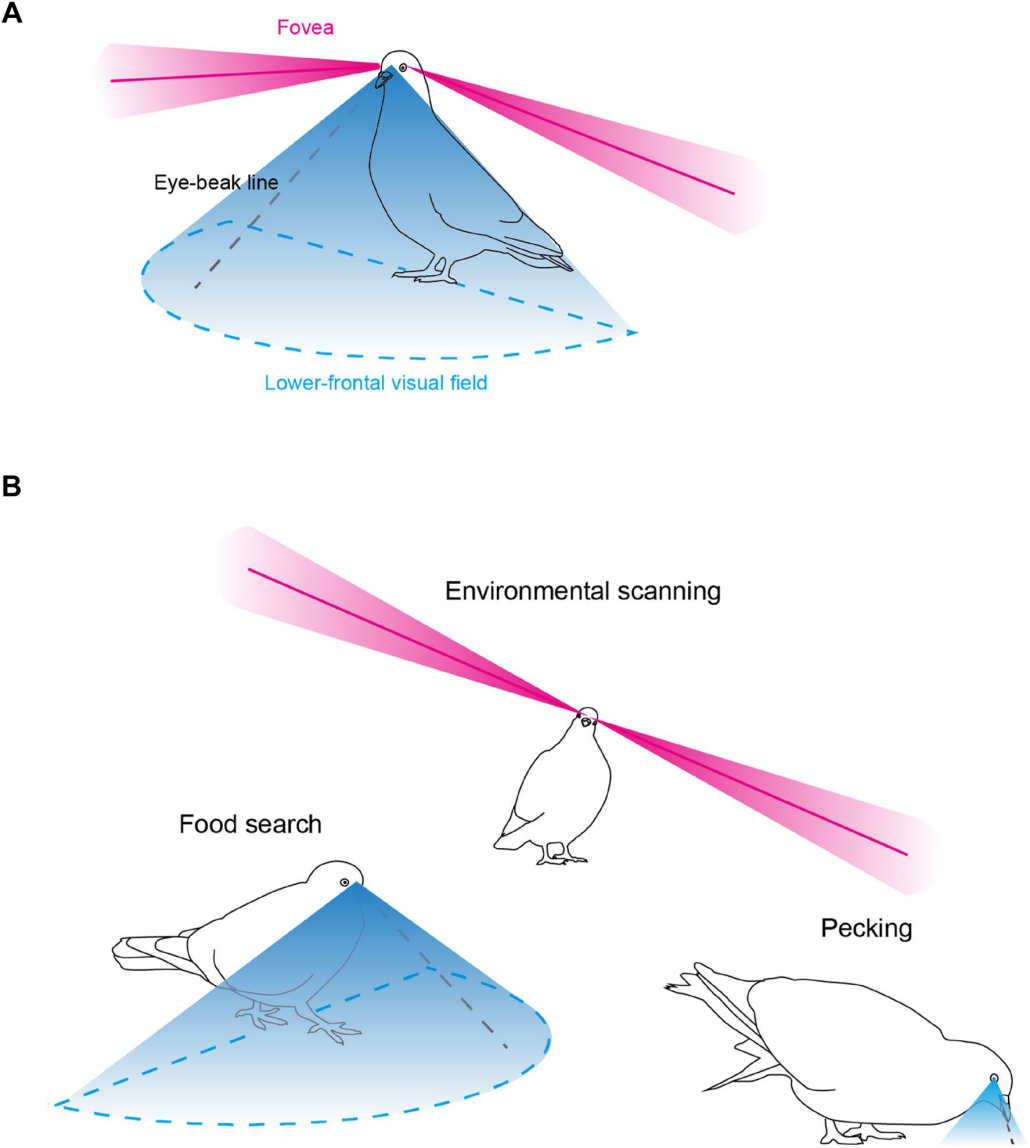
(**A**) Simple visual field model of a pigeon, assuming the projections of foveas (into the horizon) and red area (into the lower-frontal visual field). (**B**) Examples of foveal and lower-frontal visual field use across distinct behavioral contexts. Note that the omission of either foveas or lower-frontal visual field in these illustrations was made mainly for the visualization purpose, but do not necessarily suggest that pigeons use either retinal specialization exclusively at a given head angle or from a given posture. All illustrations were drawn by tracing photographs from a royalty-free image source (https://www.shutterstock.com).

The accurate reconstruction of the head-centric views of freely-moving pigeons allowed us to track the projections of their retinal specializations, foveas and red areas, and thereby to infer a few candidate locations where pigeons are attending during their free behaviors. To clarify this point, we employ a simple visual field model which defines operational ranges for such projections (Fig. 6A). As we show below, this visual field model accommodates several factors that we considered in this study, such as eye movement, distance to the visual targets, and behavioral context.

Our visual field model is based on our operational definition of visual field regions (Fig. 2c), which has proved effective in explaining various aspects of our results. Our visual field model assumes that the foveas project at an azimuth of ± 75° and an elevation of 0° (the horizon; Fig. 6A). A margin of 10° on both positive and negative sides of each fovea should well accommodate eye movement of this species^30,31^ as well as potential errors in estimating the head orientations in our system. As the foveas project into the horizon, they naturally look at a distant visual target. Notably, with the laterally projecting foveas, pigeons need to roll their head to view a distant visual target above or below their eye level (Fig. 6B); this roll movement of the head differs markedly from the pitch movement of the head that primates use in the same occasions (using their frontally projecting foveas). Our visual field model also assumes that the red areas project broadly into the lower-frontal visual field of pigeons; the azimuth from −90° to 90° and the elevation from −20° to −90° (Fig. 6B). This lower-frontal visual field looks down on the ground when pigeons are standing and walking (Fig. 6B) and is likely specialized in the search for grain in pigeons^48,49^. Relatedly, a previous study hypothesized that pigeons use motion-parallax cues to find grains on the ground during the thrust movements of head-bobbing^72^. For pecking, pigeons lower their heads and view the grain very closely (typically within 10 cm from their eyes^55^) just around their eye-beak line (Fig. 6B; also see Fig. S7). We did not assume laterality of eye use (i.e., different weights to the left and right eye) in this model because the degree of laterality was estimated overall small in this study, but this might be necessary for a certain study design. Moreover, previous studies have reported that pigeons use their upper-binocular fields to fixate on a perch during a landing flight^71^, which needs to be considered when pigeons frequently perform this behavior in a study. Although further studies are necessary to sophisticate this model, we believe that this model would help future studies to interpret pigeons’ orienting behaviors in relation to their key behaviors (e.g., feeding, vigilance), and furthermore, quantitatively estimate pigeons’ visual attention.

Lastly, it should be noted that it is not possible to specify a single attended location in pigeons (or in many other bird species) at any given moment (as it is in primate eye-tracking) because they have three projections of retinal specializations in their visual field. However, this limitation could be partly overcome by carefully designing experimental setups and analyses. For example, in an experiment, one could prepare a few candidates of visual targets that are set well apart from one another in a 3D space. Occlusion of one eye or a part of the visual field may also be effective^10^, although it may also add a handicap to the birds in natural tasks. Moreover, one could prepare sufficiently long observation periods to distinguish which candidates drew more attention. It should also be noted that pigeons have a narrow blind region and thus can perceive at least some details about a visual target in a wide range of non-foveal visual field regions. Thus, the use of visual barriers and peepholes in combination with our head-tracking system may be helpful when one needs to ensure the occlusion of their view.

In conclusion, we developed an experimental system to reconstruct the head-centric view of freely-behaving pigeons while tracking their head movement with high spatiotemporal resolution. Using this system, we have shown that pigeons primarily used their retinal specializations, fovea and red area, to view visual targets in a 3D space. Moreover, pigeons were found to “fixate” on the visual target for a relatively lengthy time when using their foveas. When a visual target was nearby (< 50 cm), however, they tended to use their red areas to view the target. Evidence for laterality of foveal use was overall limited, but observable as differences in their responses to distinct types of objects. Finally, we suggest a simple visual field model that might be useful in future studies to interpret pigeons’ orienting behaviors and quantify their visual attention. By quantifying visual attention, we expect that there will be several new opportunities to study birds’ physical and social cognition^5–8^. As our system can track the head and body movements of multiple birds in a flock, one promising avenue of future research is also the study of collective attention and behavior in a flock of birds, particularly during foraging and vigilance^18^.

## Method

We conducted 2 experiments respectively in the two study periods (August–September 2019 and November–December 2021). These two experiments were identical except that Experiment 1 presented the visual targets at a similar height to the birds while Experiment 2 presented the visual targets below or above the birds’ heads (Fig. 1D).

### Subjects

Twenty homing pigeons (*Columba livia*) housed at Max-Planck Institute of Animal Behavior, Radolfzell, participated in this study. They were originated from a local breeder, unsexed, 1–3 years old, 400–600 g in weight at the time of experiments. This number of animals was tested to detect significant differences between conditions in a within-subject design with alpha 0.05, power 0.8, and moderately strong effect sizes (> 0.65). Ten birds participated in each of the two experiments. Birds roost in a loft as a flock (in one of the 5 compartments sized 2w × 2d × 2 h m, where w, d, and h denote width, depth, and height, respectively). Birds have ad-lib access to water and are fed grains once a day. On the day of the experiments, they were brought from the loft in a carrier box (60w × 35d × 23 h cm) to the mo-cap room, and after the experiments (it took typically 1–2 h), they were brought back to the loft and fed additional grains. The experimental procedures of this study were performed in accordance with the German regulation on animal experimentation and approved by the Ethical Committee of Baden-Württemberg (Regierungspräsidium Freiburg, Referat 35, License Number: 35-9185.81/G-19/107). We report all methods in accordance with ARRIVE guidelines.

### Apparatus

Pigeons wore custom backpacks (made of lightweight fabrics and elastic bands, 3 g in total^59^) throughout the study period. Pigeons were habituated to wearing backpacks before the experiments. Before a daily experiment, four 6.4-mm (in diameter) reflective markers (together < 1 g) were attached to the feathers of a pigeon’s head with double-sided tapes and glue. Additionally, a Styrofoam plate (3.5w × 7d cm) with four 9.5-mm markers on marker bases (5–10 mm height) was attached to the backpack with Velcro (together < 5 g). Attachments to pigeons weighed less than 10 g, which was less than 2–3% of the weights of our pigeons (on average, they weighed 500 g). No habituation was necessary for pigeons to wear head markers because all pigeons generally tolerated them. All head markers and the Styrofoam plates were removed from the pigeons after the daily experiment (and reattached on the next experimental day). The 3D coordinates of the head and body markers of pigeons were recorded at 100 Hz and with submillimeter precisions in a motion-capture system (15w × 7d × 4 h m). This system was equipped with 30 high-resolution infrared cameras (VERO/VANTAGE, VICON, Oxford, UK) in addition to four optic cameras (VUE, VICON). All cameras were automatically synchronized. The system was calibrated with a calibration wand before each trial.

### Head-orientation calibration

The head-centric coordinate system was reconstructed based on 3D coordinates of head markers and morphological key points, particularly the beak tip and the center of each eye. To align the coordinates of head markers and those of morphological key points, we developed two calibration methods respectively in the two experiments. While both calibration methods were comparable in their accuracies (see below), we critically improved the efficiency of procedures in the second calibration method, which reduced the time required for handling pigeons during the head calibration. In the first calibration method, the head of each pigeon (with the head markers) was lightly held by an experimenter, and the custom calibration grid with four 9.5-mm markers was visually aligned to the two eyes and the beak tip by another experimenter seeing these features through small mirrors (Fig. S1). The second calibration employed digital rather than manual procedures. As shown in Fig. 1B, the pigeon’s head was filmed from four distinct angles by standard webcams (C270, Logitech, Lausanne, Switzerland) synchronized in a capture software (MultiCam Capture, Corel, Ottawa, Canada). A triangular scale (2 cm on each side) was attached temporarily to the root of the pigeon’s beak during the calibration (and then removed after the head calibration) to give information about an absolute scale (length) to the system. The distortions of captured images were corrected using a built-in MATLAB application (“Camera Calibrator”, MATLAB, MathWorks, Natick, USA). We then manually identified the (2D) coordinates of marker centers, and key morphological points, i.e., eye centers and beak-tip, in captured images (this process can be automated in future studies, using a deep-learning-based method) and then reconstructed the 3D coordinates using our custom software which employed the MATLAB structure-from-motion functions, which is available in an online repository (see the data availability statement). This latter method has been developed and validated in a previous study^73^.

Based on the obtained 3D coordinates, we defined the head-centric coordinate system with its origin located at the midpoint of two eyes, its Y-axis parallel to the horizon and pointing to the beak tip, its X-axis parallel to the horizon and pointing to the right eye, and Z-axis orthogonal to X and Y axes (Fig. 1C). The horizon is referenced in the head-centric coordinate system because it is a meaningful dimension in our behavioral experiments. The beak of pigeons points down about -30° in pitch with respect to the horizon in natural standing/walking postures (Fig. S4, also see^74^). We defined the horizon as the transverse plane of a pigeon’s head in each recording; thus, for each recording, we calculated the single median value of roll angles (around 0°) and that of pitch angles (around −30°; see Fig. S4 and also see^74^) from all frames and then rotated each bird’s head orientations by those median values respectively for roll and pitch in all frames. It should be noted that, although we performed these corrections for each recording in this study because we were concerned with the individual variations in natural standing/walking postures and head calibration errors, these corrections can be omitted/simplified in future studies because those variations/errors were observed to be small in this study (specifically, the correction of the roll angles can be omitted, and the head can be rotated in pitch by a constant value of −30° for all frames).

We estimated the accuracies of our calibration methods by repeating the above procedures ten times for each calibration procedure using a bird’s head model equipped with four 6.4-mm markers, eyes, and beak-tip in specified locations. We found that the mean errors (the difference between the estimated and measured coordinated system) were 0.38° ± 0.24°, 0.50° ± 0.34°, 0.74° ± 0.55° (mean ± SD) respectively in yaw, roll, and pitch for the first calibration method and 0.27° ± 0.12°, 0.32° ± 0.21°, 0.60° ± 0.25° respectively in yaw, roll, and pitch for the second calibration method (1° calibration error corresponds to a 1.7 cm error at a one-meter distance). It should be noted that the errors could be slightly larger in real pigeons because the key morphological features, namely the center of the eye and beak tip, were typically harder to be identified than in the model.

### Stimuli and procedure

Each experimental day started with the attachment of reflective markers to the pigeons (3–5 min per bird), followed by the head calibration (2–3 min per bird in the first method, and less than a minute per bird in the second method). We attached the head and backpack markers each experimental day and removed them soon after the daily experiment (this daily removal and reattachment of markers was performed mainly for ethical reasons in order to allow birds to freely behave in their loft without any marker attachment after the daily experiment). They were then released into the motion-capture room and allowed to move freely during the trial (Table S2 for the detailed procedures each day). After 2–3 min of acclimatization, we presented to them attention-getting visual targets at various relative locations (Fig. S2). We presented a visual target to both single and multiple (2 or 4) pigeons (all pigeons were focals in the latter case); we included both types of recording in our analyses because the results from those recordings were largely similar.

We prepared three types of visual targets; Type-1 (moderately threatening stimuli), Type-2 (detailed stimuli), and Type-3 (social stimuli). In Experiment 1, a Type-1 visual target was a medium-sized object with a variety of colors and shapes (approximately 7w × 5d × 4 h cm, with five 9.5-mm makers) and slid on the ground toward the pigeon in Experiment 1. The Type-2 visual target was a peanut (about 1 cm in diameter) attached to a 9.5-mm marker and thrown to the pigeon alternately with peanuts (with no marker attachment) when the pigeon was standing/walking on the ground. The Type-3 visual target was a conspecific released to the motion-capture room while the focal bird (already in the room) was standing/walking on the ground. In Experiment 2, a Type-1 visual target was a looming image presented repeatedly on a smartphone (60 Hz, Google Pixel 2, Googleplex, Mountain View, USA; approximately 15w × 7d cm, with five 9.5-mm makers). The experimenter held the smartphone still and oriented the screen toward the focal bird at a height of about 1.3 m from various distances (2–4 m; Fig. S2). The looming image was created in Premiere Pro (Adobe, Mountain View, USA) and made of various sorts of dark-colored simple geometry or predator silhouette in a gray background. The Type-2 visual target in Experiment 2 was a small object (5–10 cm in diameter) of a variety of colors and shapes thrown to the ground alternately with a variety of grains when the pigeon was walking/standing on a table (200w × 30d × 75 h cm); in Experiment 2, the presented object was larger than the one used in Experiment 1 (peanut attached to a marker) because our pilot trials (see below) confirmed that the latter object did not seem to attract the birds’ attention sufficiently at a larger visual distance in Experiment 2 (due to the elevation of the table); our pilot trials also confirmed that those larger objects did not elicit the birds’ evasion responses (unlike Type-1). The Type-3 visual target in Experiment 2 was a conspecific released to the motion-capture room while the focal bird (already in the room) was standing/walking on the table. In both experiments, a Type-1 or Type-2 object was replaced with another object at each presentation event to maximize the birds’ attention to the visual targets. In both experiments, we threw these Type-1 and Type-2 objects toward the focal pigeon independently of their postures and tried to make the objects land as closely as possible to the pigeon (without hitting the pigeon). Type-3 stimulus (a conspecific) typically walked or flew toward the focal pigeon soon after the release. Before each experiment, we performed several pilot trials with 2–4 pigeons to explore the presentation conditions that likely maximize their spontaneous orienting responses to the presented small objects/foods (by direct observation); those pigeons also participated in each experiment, but we did not find different patterns of results in those pilot subjects compared to the others and thus included all subjects into our final analyses.

Each bird was tested for 6–9 days in Experiment 1 (7.5 ± 0.85, mean ± SD) and 6 days in Experiment 2. Each day consisted of several short recordings, and each recording presented several visual targets one by one (see Table S2 for the details). We dropped 3% of all recordings due to recording failures or experimental errors. The order of testing each bird and the combination of birds tested each day was counterbalanced across days.

### Data analysis

#### Motion-capture data processing

In the motion-capture software (NEXUS 2, Vicon), we first defined the rigid bodies respectively for the bird’s head and backpack (on its body) from the unique arrangements of the attached markers in a single frame. Additionally, we connected the rigid bodies of the head and backpack with a free joint and defined it as a single skeleton. Each marker was then automatically labeled for all frames in the software. The head markers were often mislabeled by the software within the head’s rigid body, leading to erroneous rotations of the head. We corrected this error in our custom MATLAB codes by first identifying the time frames in which the distance between the head markers abruptly changed (larger than 5 mm in adjacent frames) and then permuting the marker labels in each section (between the frames detecting the abrupt changes) until such abrupt changes were eliminated in the entire recording. It should be mentioned that such a mislabeling issue could emerge in any future study with a motion-capture system, but it critically depends on the resolution of motion-capture cameras, the size of each marker, the configuration of the markers and the skeleton, and the algorithms employed by the motion-capture software. To aid future studies to solve the same issue, we post our custom MATLAB codes in our online repository (see the data availability statement). The markers on the visual target objects were also labeled in the motion-capture software, either automatically (for Type-1 objects having several markers) or manually (for Type-2 objects having only one marker). Finally, the global coordinates of visual targets were converted into the local coordinates in the bird’s head-centric coordinate system.

#### Data extraction

We then extracted the moments in which the focal birds likely oriented to a visual target in our custom MATLAB codes (available in our online repository). For the visual targets thrown/slid to the focal pigeon (Type-1 and 2 targets in Experiment 1 and Type-2 targets in Experiment 2), we extracted 2 s from the moment in which the visual target just stopped (< 0.2 m/s). In Experiment 2, we excluded the moments in which the target was visually occluded from the focal’s eyes by the table; if this exclusion made the duration of the event (maximum 2 s) shorter than 0.5 s, we excluded that event from the analysis. The total observation time per bird (including saccadic moments) was 112.9 ± 31.8 s (mean ± SD) for Type-1 targets and 220.6 ± 31.5 s for Type-2 targets in Experiment 1 and 168.7 ± 11.1 s for Type-2 targets in Experiment 2. For the looming images (Type-1 targets in Experiment 2), we analyzed the moments in which the images were presented to the pigeons. The total observation time per bird was 426.2 ± 74.2 s (mean ± SD) for this visual target. For the conspecific targets (Type-3 targets in Experiments 1 and 2), we analyzed the first 30 s after another pigeon entered the motion-capture room while the focal pigeon was already present. In Experiment 2, we extracted the moments in which the focal birds were on the table and the target birds (just entering the mo-cap room and walking toward the table) were on the ground. Additionally, we excluded the moments in which the target bird was visually occluded from the focal’s eyes by the table in both Experiments 1 and 2. The total observation time was 183.9 ± 37.5 s (mean ± SD) in Experiment 1 and 60.9 ± 36.2 s in Experiment 2.

#### Data filtering

We performed data filtering using our custom MATLAB codes (available in our online repository), following the steps detailed in Fig. S8. Briefly, we first filtered and exported the 3D coordinates of markers in the motion-capture software. We then imported the data into our custom software, converted the 3D coordinates into both translational and rotational movements of the head and the translational movements of the back, and visual targets, and further filtered them. The gaze data were smoothed at 30 Hz (this frequency was largely sufficient to detect head saccades in our data). Our choices of parameters were described in Fig. S8. We determined those parameter values by visually inspecting all data to minimize any impossible movement (both biologically and physically) and also to minimize the missing values in the time series of data. In addition to these movement filters, we additionally filtered out the moments in which birds made head saccades (19.5% of all data) because visual processing is likely inhibited during saccades in birds^75^. Head-bobbing consists of “thrust and hold” phases in pigeons and could be identified as the acceleration of translational (rather than rotational) head movement ^72,76^. Although the thrust movement of the head is also ballistic similar to head-saccade, we did not exclude this phase from our data because a previous study indicated that visual processing is not inhibited during this phase in pigeons^46^.

#### Saccade detection

A head-saccade was detected in frames in which the axial angle speed exceeded 60°/s^11^ (Fig. S6). This axial angle was calculated from the rotation matrix that converts the head angle recorded in a given time frame to that recorded in the next frame (this axial angle was very close to √(*y*^2^ + *r*^2^ + *p*^2^), where *y*, *r,* and *p* respectively denote yaw, roll, and pitch, obtained from the same rotation matrix). Although there was a population of small saccades (shorter than 50 ms and smaller than 5°) in our data, which was consistent with a previous study^11^, we excluded them from our analyses (thus included them in the inter-saccadic intervals) because they were not distinguishable from the motion-capture noises in our data.

### Statistical analysis

We summarized our statistical approach in Table S3. For the first two analyses (the birds’ use of different visual field regions), we used linear mixed models (LMM) with Gaussian error structure and identity link function using “lme4” in R (ver. 4.0.5). The response variable of the first analysis (the proportion of time per nonempty bin) was calculated as the proportion values and then logit-transformed to be tested in LMM. We could not use non-proportion values in this particular analysis (unlike our other analyses) because we excluded the empty bins from the data to calculate the proportion values (i.e., the variable could not be represented as the number of hit/miss); we thus logit-transformed the proportion values according to the recommendation by Warton and Hui^70^. We checked the assumptions of normally distributed and homogeneous residuals in diagnostic plots; namely, histogram of residuals, Q–Q plots of residuals, and residuals plotted against fitted values. We checked the model stability by removing each level of each random factor (subject, presentation condition) one by one and calculating Cook’s distance each time (“influence.Me”), which revealed that one presentation condition (presenting Type-1 object above the birds’ head) was potentially influential in the first two analyses (as also noted in “Results and discussion”). We confirmed that removing this condition does not affect the results in both analyses. For the third analysis (the effect of distance), we used generalized mixed models (GLMM) with binomial error structure and logit link function. We checked overdispersion using the dispersion parameter derived from “blmeco” and did not find it to be an issue. In all of the above analyses, random effects structures were kept maximal (including both random intercepts and all possible random slopes). We tested significance using a likelihood ratio comparing the full model with a model without the effect in question (“anova”). For the final analyses (laterality), we used LMM with the same model structure after confirming the same model assumptions. All processed data and R codes are available in our open data repository (see the data availability statement).

## Supplementary Information

Supplementary Information.

Supplementary Video 1.

## Data Availability

All processed data, R codes for the statistics, the MATLAB codes for the data processing, and the sample data are available in OSF (https://osf.io/zuthk/?view_only=d13ee480ef3b48a0b85e9818e4f06bb3). The head calibration application is available in GitHub (https://github.com/itaharaakihiro/head_orientation_calibration_app_set).
