# Supplementary material for: Head-tracking of freely-behaving pigeons in a motion-capture system reveals the selective use of visual field regions
Source: Sci Rep. 2022 Nov 9;12:19113. doi: 10.1038/s41598-022-21931-9 (PMC9646700; doi:10.1038/s41598-022-21931-9)
Supplement: Supplementary file 1 — Supplementary Information. [file 41598_2022_21931_MOESM1_ESM.pdf]

Supplemental materials: Head-tracking of freely-behaving pigeons in a motion-capture system reveals the selective use of visual field regions

Fumihiro Kano, Hemal Naik, Göksel Keskin, Iain D. Couzin, Mate Nagy

Table S1. Summary of experimental conditions and procedures

Experiment	N of subjects	Object type	Object content	Object position	Object time window	Study period	Head calibration method
**Exp 1**	10	Type-1 (moderately threatening)	An approaching object	Slightly below the birds' eye level	0-2 sec. after the object stopped	Aug-Sep 2019	Manual with a head-calibration grid
		Type-2 (detailed)	Grain attached to a reflective marker	Slightly below the birds' eye level	0-2 sec. after the object stopped		
		Type-3 (conspecific)	A conspecific just released	At the birds' eye level	0-30 sec. after the conspecific appeared		
**Exp 2**	10	Type-1 (moderately threatening)	A looming image on a small monitor	Above the birds' eye levels	Cumulatively about 1 min.	Nov-Dec 2021	Digital with a head-calibration frame
		Type-2 (detailed)	A small object thrown alternately with grain	Far below the birds' eye level	0-2 sec. after the object stopped		
		Type-3 (conspecific)	A conspecific just released	Far below the birds' eye level	0-30 sec. after the conspecific appeared		

Table S2. The procedures of testing each bird each day. Each column shows the recording ID, the focal bird, the stimulus presented to the focal bird, the bird(s) released into the mo-cap room before the recording, the bird(s) retrieved from the mo-cap room after the recording, and additional notes.

Recording ID	Focal bird	Stimulus presented	Releasing (before the recording)	Retrieving (after the recording)	Note
**Recording 1**	Bird A	Type 2	Bird A		
**Recording 2**	Bird A	Type 1		Bird A	
**Recording 3**	Bird B	Type 2	Bird B		
**Recording 4**	Bird B	Type 1			
**Recording 5**	Bird B	Bird A	Bird A		
**Recording 6**	Bird A, B	Type 2			
**Recording 7**	Bird A, B	Type 1		Bird A and B	Performed only in Exp. 2
**Recording 8**	Bird C	Type 2	Bird C		
**Recording 9**	Bird C	Type 1		Bird C	
**Recording 10**	Bird D	Type 2	Bird D		
**Recording 11**	Bird D	Type 1			
**Recording 12**	Bird D	Bird C	Bird C		
**Recording 13**	Bird C, D	Type 2			
**Recording 14**	Bird C, D	Type 1			Performed only in Exp. 2
**Recording 15**	Bird C, D	Bird C, then Bird D	Bird C, then Bird D		
**Recording 16**	Bird A, B, C, D	Type 1		Bird A, B, C, D	Performed only in Exp. 1

*Type-1 (moderately threatening) targets were presented 5-8 times, and Type-2 (detailed) targets were presented 8-15 times in each recording
*Each recording lasted for 2-3 min, and the entire recording took about 1 hour.
*Some recordings (7, 14, 15) were skipped in Exp. 1 or 2 due to the time constraints each day.

Table S3. Summary of our statistical approach.

Test	Response	Fixed factor (Number of levels)	Random factor (Number of levels)	Model type
**Differential use of visual field regions**	Mean proportion of time per nonempty bin (logit-transformed)	Visual field region (5)	Subject (20), Presentation condition (6)	LMM
**Differential use of visual field regions**	Intersaccadic interval (sec)	Visual field region (5)	Subject (20), Presentation condition (6)	LMM
**Effect of distance on the use of foveal and lower-frontal regions**	Number of frames in which the visual target was hit/missed	Distance bin (16), Visual field region (2)	Subject (20)	Binomial GLMM
**Laterality of foveal use in response to different object types**	Laterality score	Object type (3)	Subject (20)	LMM

*LMM – Linear Mixed Model, GLMM, Generalized Linear Mixed Model

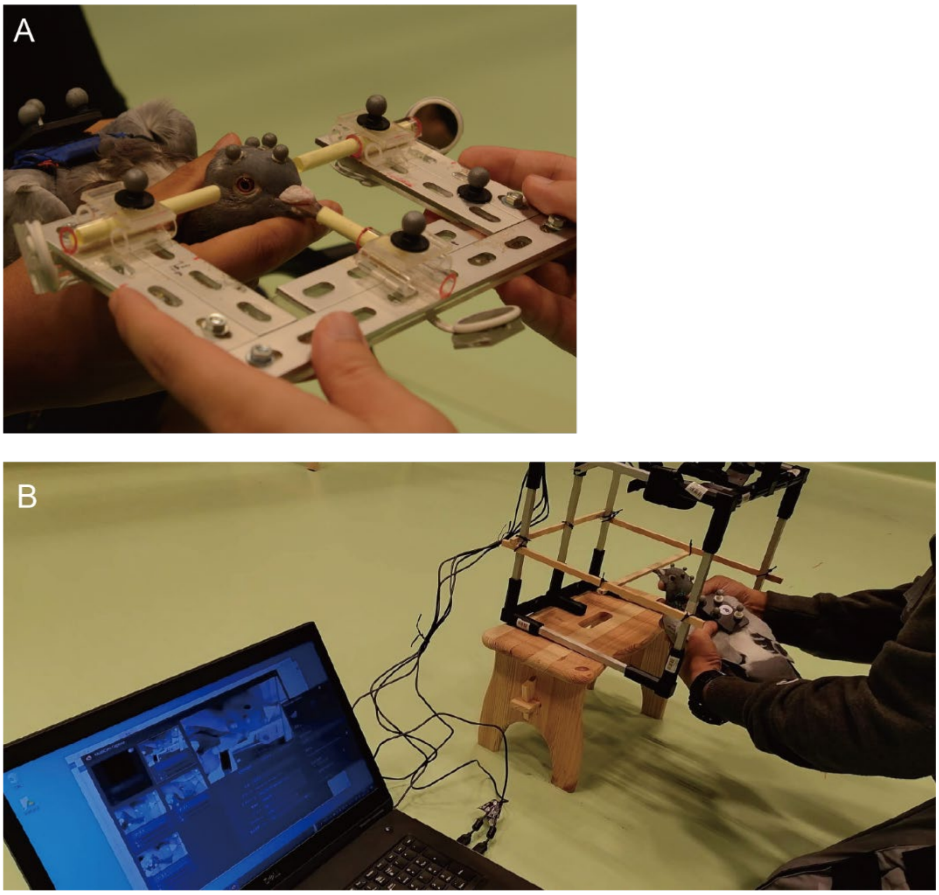

Figure S1. Two head calibration methods in this study. A. Calibration grid with mo-cap markers. B. Multi-camera calibration volume.

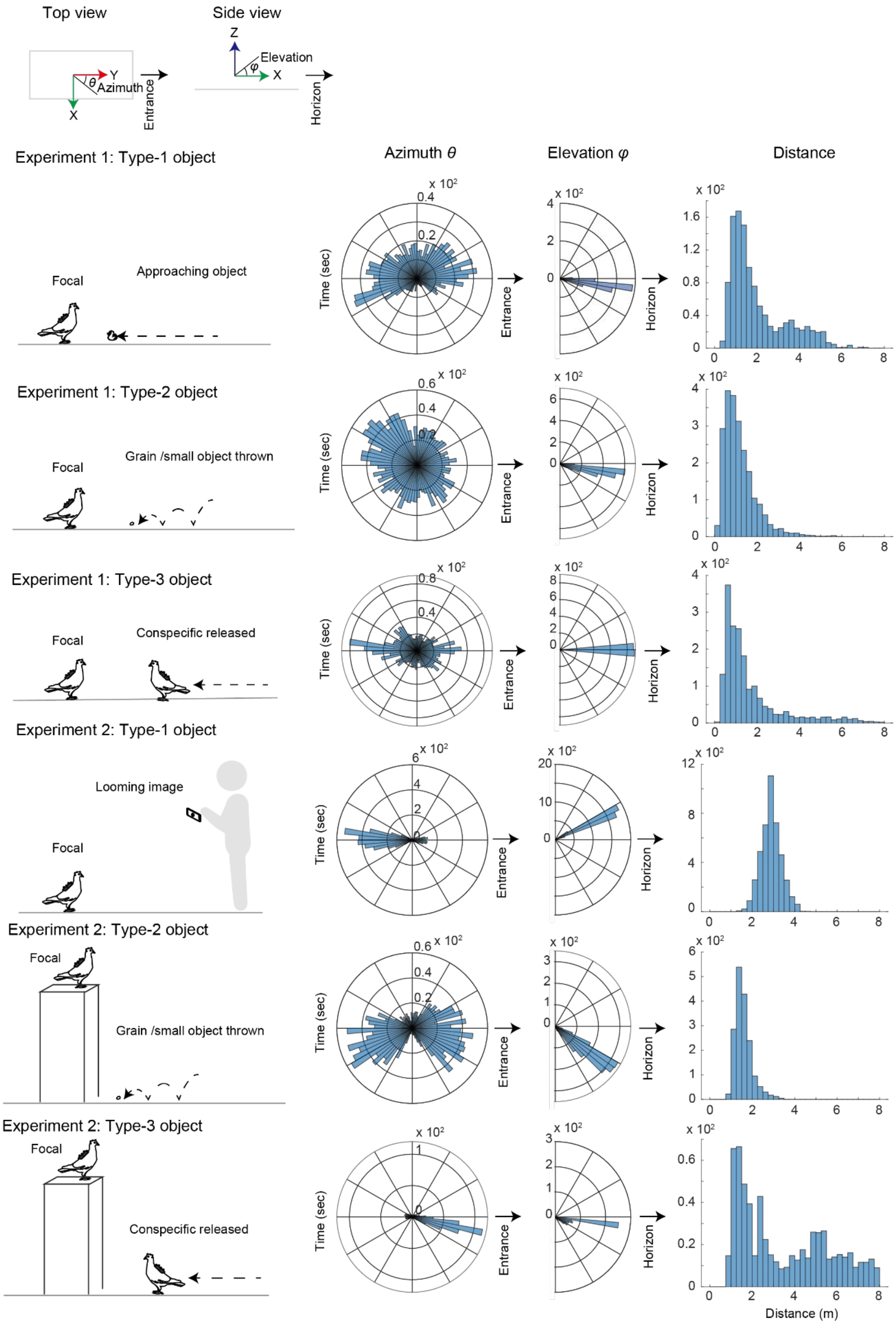

Top view
Side view
Y
θ Azimuth
X
Entrance
Z
Elevation
φ
X
Horizon
Experiment 1: Type-1 object
Azimuth θ
Elevation φ
Distance
Focal
Approaching object
Experiment 1: Type-2 object
Focal
Grain /small object thrown
Experiment 1: Type-3 object
Focal
Conspecific released
Experiment 2: Type-1 object
Looming image
Focal
Experiment 2: Type-2 object
Focal
Grain /small object thrown
Experiment 2: Type-3 object
Focal
Conspecific released
Time (sec)
x 10²
Distance (m)

Figure S2. Distribution of the presented visual targets in the motion-capture room across the two experiments. The Polar coordinates of visual targets were shown as the azimuth, elevation, and distance in the coordinate system with its origin located at the focal pigeon's head (the center of the two eyes), its Z-axis oriented to the zenith, and its Y-axis oriented to the entrance of the mo-cap room and the horizon.

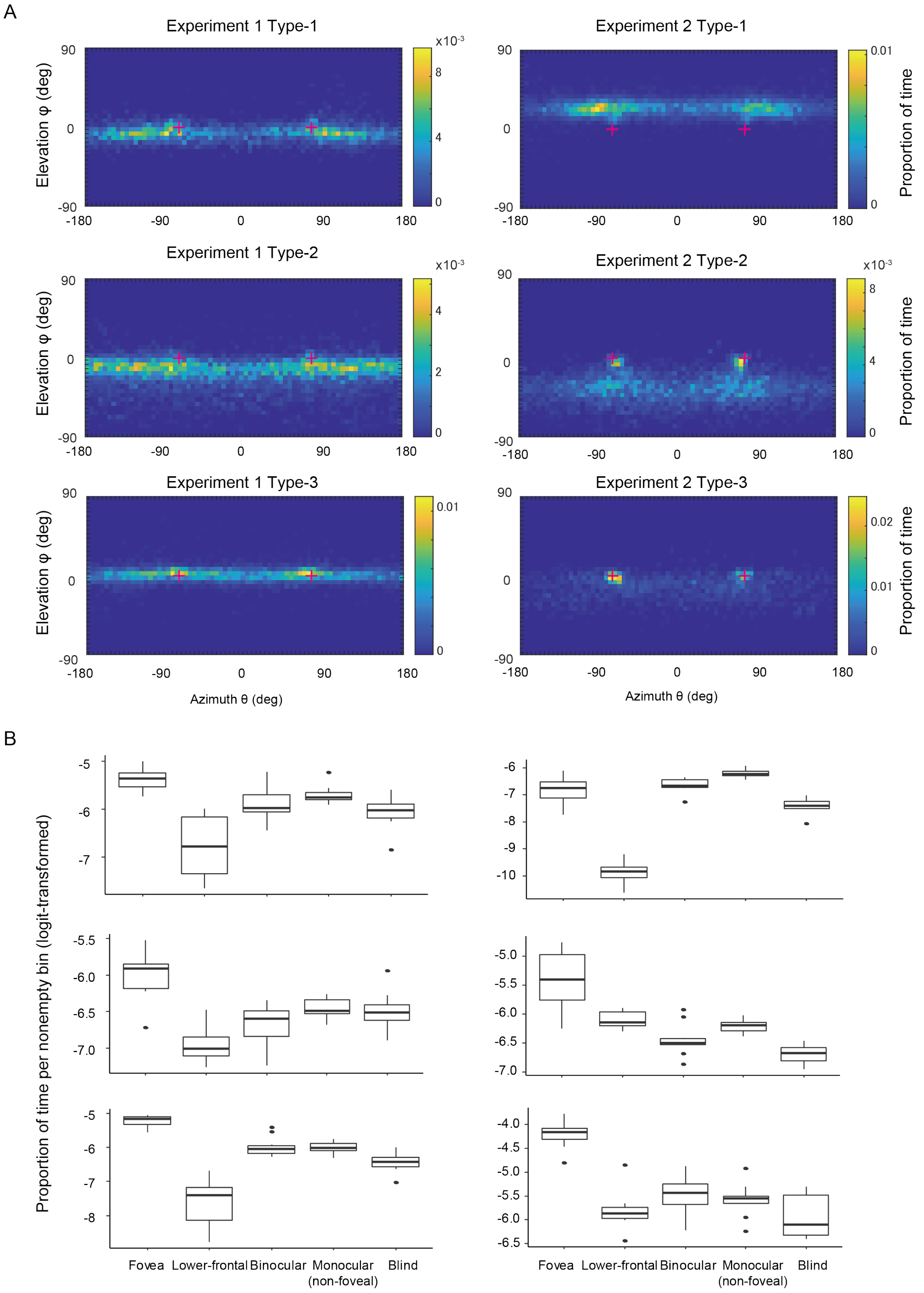
A
Experiment 1 Type-1
Experiment 2 Type-1
Experiment 1 Type-2
Experiment 2 Type-2
Experiment 1 Type-3
Experiment 2 Type-3
Elevation φ (deg)
Azimuth θ (deg)
Proportion of time
x10⁻³
B
Proportion of time per nonempty bin (logit-transformed)
Fovea
Lower-frontal
Binocular
Monocular (non-foveal)
Blind

Figure S3. A. The distribution of visual targets in pigeons' visual field in each experiment per object type. Each bin was sized 5° x 5° and indicate the proportion of time during which visual targets were observed in this bin. Also shown is the combined map for all experiments. The cross marks indicate the location of foveas in the visual fields (at an azimuth of ±75° and an elevation of 0°). B. Proportion of time during which visual targets were observed in each non-empty bin of each visual field region (logit-transformed), separated for each experiment and object type. Box plots show the median, interquartile range (IQR), and 1.5 × IQR with outliers plotted individually.

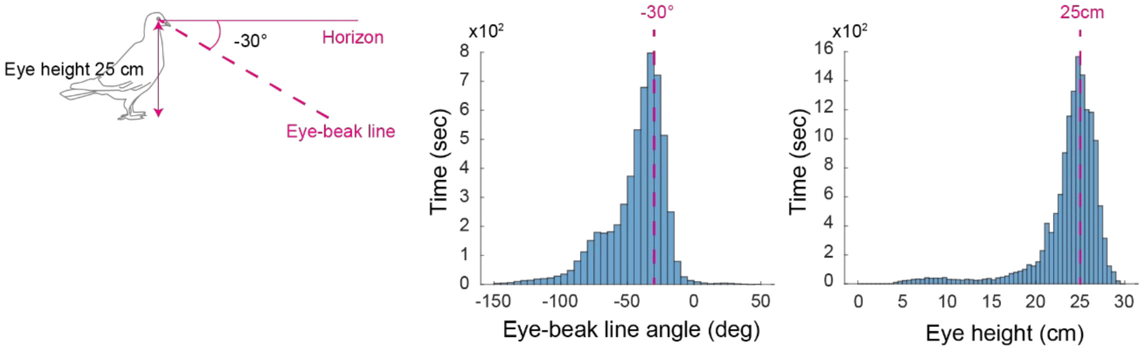

Figure S4. Frequency of the elevation angle of the beak (per 5° bin) in the head-centric coordinate system and the eye height from the ground (the midpoint of the two eyes; the origin of the head-centric coordinate system). The latter data included only the moments when the birds were on the ground (0 cm) but not on the table (73 cm). We derived these data from Experiment 2 because the calibration method used in Experiment 2 identified the eye centers and beak tip more directly than that used in Experiment 1.

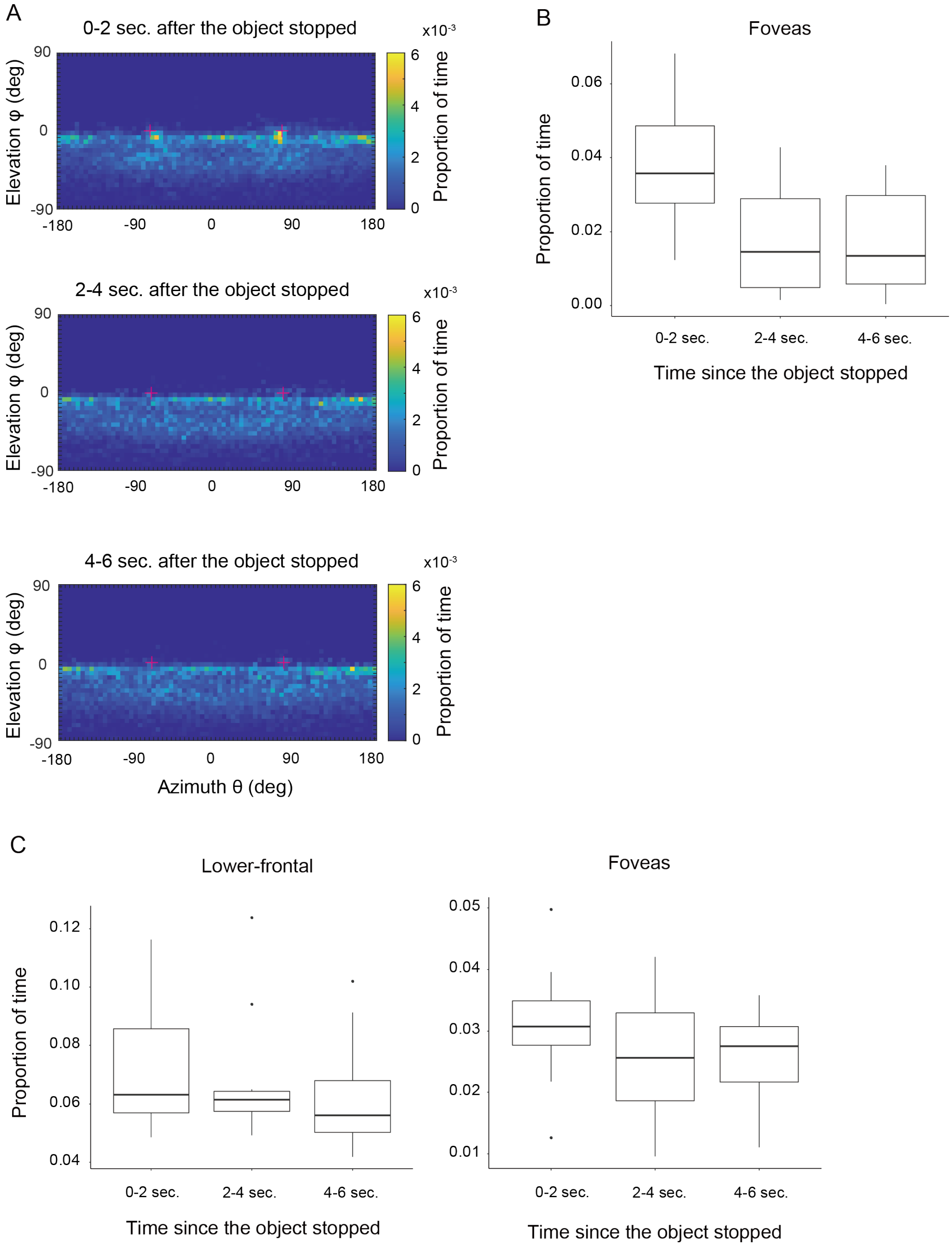

Figure S5. A. The distribution of visual targets in pigeons' visual field in each experiment as a function of time since the presented object (Type-1 and -2 objects in Exp. 1 and Type-2 objects in Exp.2) stopped its movements (in 2-sec bins). Note that we assumed that the initial time

window (0-2 sec.) captures pigeons' attention most strongly and thus used this time window in our main analyses. In this figure, we show how pigeons altered their attention to the visual targets after this initial time window. Each bin was sized 5° x 5° and indicate the proportion of time during which visual targets were observed in this bin. The cross marks indicate the location of foveas in the visual fields (at an azimuth of ±75° and an elevation of 0°). B. Proportion of time per bird during which visual targets were observed in the foveal region as a function of time. To test the effect of time, we performed a binomial GLMM with the number of frames in which the visual target was hit or missed in the foveal region as a response (i.e., the proportion of time), the time bin a fixed factor, and the subject as a random factor. We found a significant effect of the time bin (Likelihood ratio test, $\chi^2(2) = 16.22$, $P = 0.0003$). C. The same analysis on the condition where Type-2 (detailed) objects were presented in Experiment 1; note that this analysis is related to the analysis of the effect of distance in the main texts (Figure 4). To test the effect of time on the pigeons' use of frontal and foveal visual field areas, we performed GLMM identical to the above except that it has the time window, the visual field region, and their interaction as fixed factors. After the removal of a nonsignificant interaction term ($\chi^2(2) = 3.50$, $P = 0.17$), we found a significant effect of the visual field region ($\chi^2(1) = 15.63$, $P < 10^{-4}$), and importantly, a significant effect of the time window ($\chi^2(2) = 6.25$, $P = 0.044$).

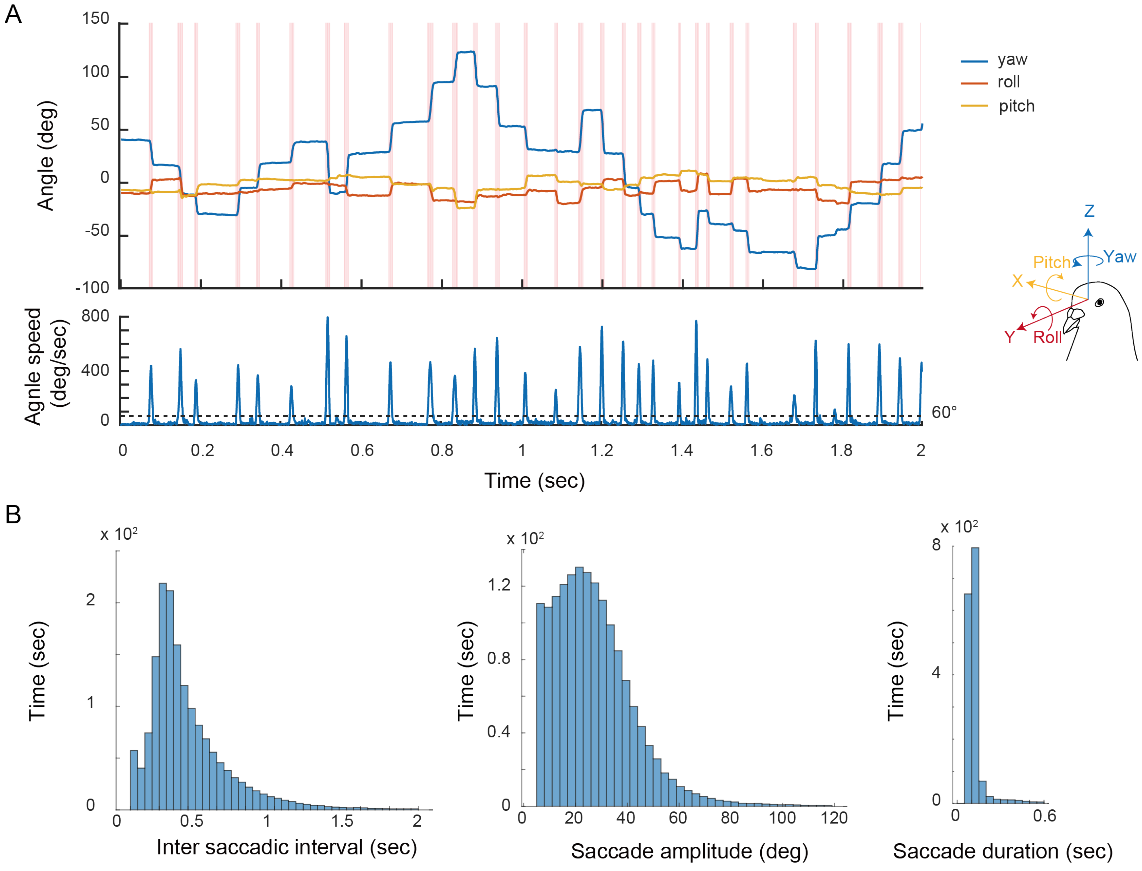

Figure S6. A. Example of head movement in yaw, roll, and pitch. Saccades were highlighted in magenta. The axial angle speeds are shown in the bottom panel. B. Histograms of intersaccadic interval (sec), saccade amplitude (deg), and saccade duration (sec).

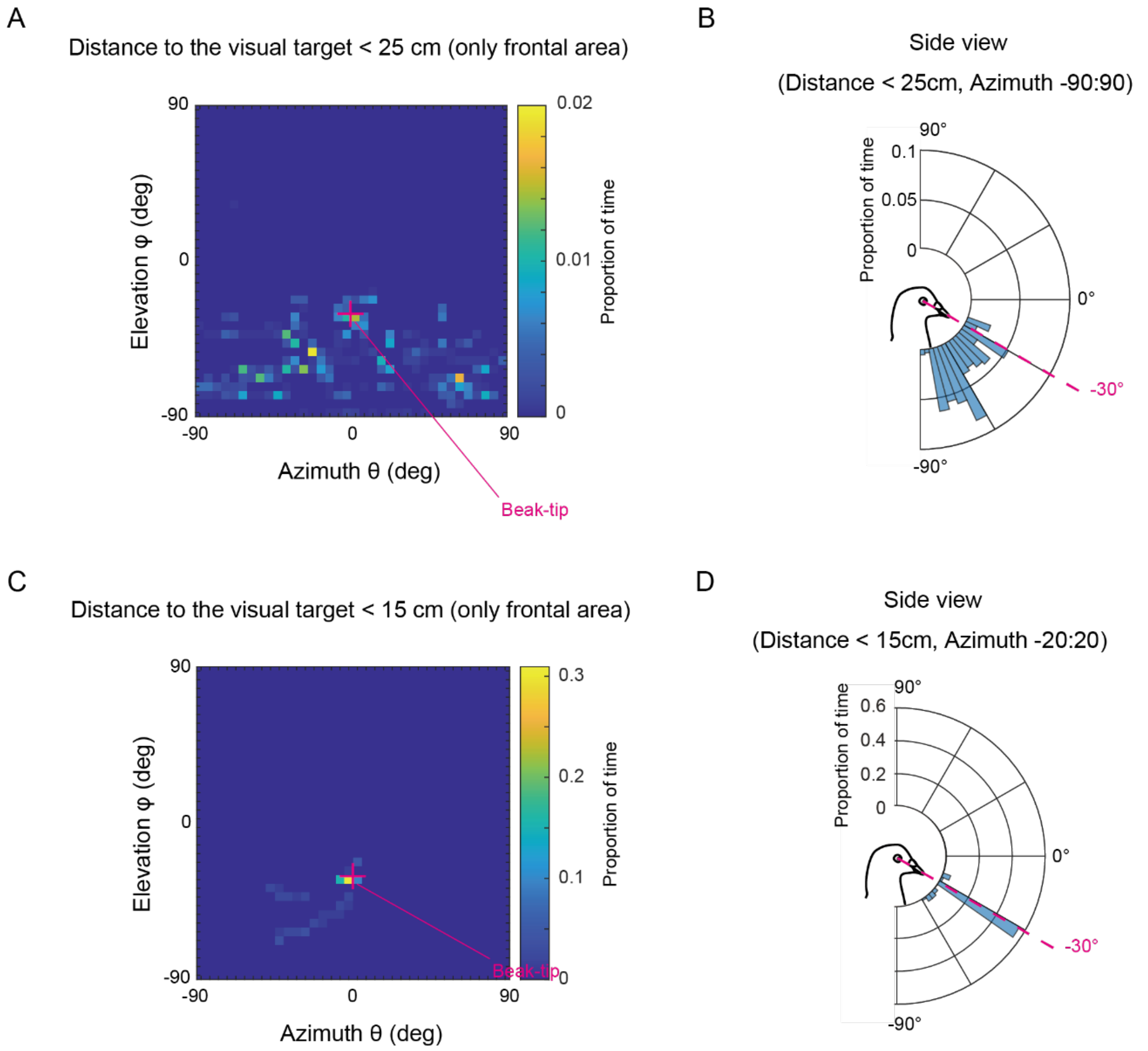

Figure S7. A. The distribution of visual targets (the proportion of time during which visual targets were observed in each 5°x5° bin) when the visual targets were in front of the pigeons' heads (at the azimuth from -90° to 90°) and closer than 25 cm (when the pigeons slightly lowered their head) in Experiment 1 presenting Type-2 targets (detailed stimuli). The cross mark indicates the coordinate of the beak tip. B. The same data in a Polar histogram (the side view). C. Identical to A except that the visual targets were closer than 15 cm (when the pigeons were prepared to peck it). D. The same data in a Polar histogram (the side view), while restricting (for the visualization purpose) the distribution to a range including the azimuth from -20° to 20°.

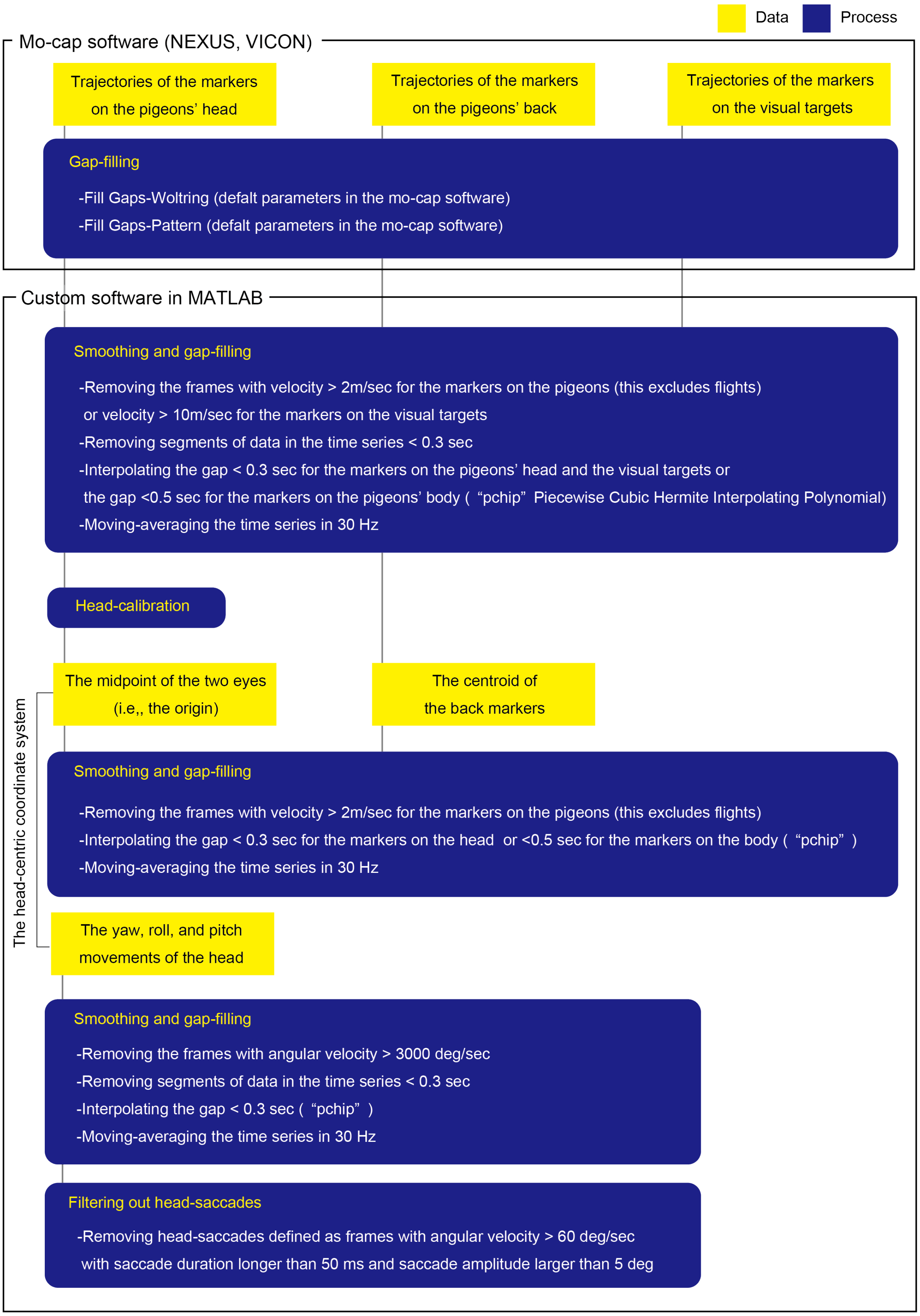

Figure S8. The data-filtering procedure. Trajectories of markers were first gap-filled in the

motion-capture software and then cleaned, again gap-filled, and smoothed in the custom software. These trajectories were then converted to the centroids and coordinate system and further cleaned, gap-filled, and smoothed in the custom software. Finally, head saccades were filtered out from the yaw, roll, and pitch movements of the head.

Video S1. Examples of experimental recordings with reconstructed "gaze". https://youtu.be/jlTCNdSN75M
